# Genetics of ischemic stroke functional outcome

**DOI:** 10.1007/s00415-024-12263-x

**Published:** 2024-03-19

**Authors:** Troy P. Carnwath, Stacie L. Demel, Charles J. Prestigiacomo

**Affiliations:** 1 University of Cincinnati College of Medicine, Cincinnati, OH 45267 USA; 2 Department of Neurology, University of Cincinnati College of Medicine, Cincinnati, OH 45267 USA; 3 Department of Neurosurgery, University of Cincinnati College of Medicine, Cincinnati, OH 45267 USA

**Keywords:** Genomics, Genetics, Ischemic stroke, Stroke outcome, Molecular pathophysiology, Precision medicine

## Abstract

Ischemic stroke, which accounts for 87% of cerebrovascular accidents, is responsible for massive global burden both in terms of economic cost and personal hardship. Many stroke survivors face long-term disability—a phenotype associated with an increasing number of genetic variants. While clinical variables such as stroke severity greatly impact recovery, genetic polymorphisms linked to functional outcome may offer physicians a unique opportunity to deliver personalized care based on their patient’s genetic makeup, leading to improved outcomes. A comprehensive catalogue of the variants at play is required for such an approach. In this review, we compile and describe the polymorphisms associated with outcome scores such as modified Rankin Scale and Barthel Index. Our search identified 74 known genetic polymorphisms spread across 48 features associated with various poststroke disability metrics. The known variants span diverse biological systems and are related to inflammation, vascular homeostasis, growth factors, metabolism, the p53 regulatory pathway, and mitochondrial variation. Understanding how these variants influence functional outcome may be helpful in maximizing poststroke recovery.

**Supplementary Information:**

The online version contains supplementary material available at 10.1007/s00415-024-12263-x.

## Introduction

Stroke is the leading neurological cause of disability-adjusted life years, globally [89]. Every year in the United States, nearly 800,000 people suffer from a new or recurrent stroke with 13.6% of patients expiring, making cerebrovascular accidents the 5th most common cause of death [277]. Fortunately, stroke incidence and mortality are declining, the latter steadily decreasing since the early 1900s, and more notably in the last four decades [150, 156]. Nonetheless, our aging population bears an increased lifetime risk of stroke, rising from 22.8% in 1990 to 24.9% in 2016, with a relative risk increase of 8.9% [336]. Considering that 50% of stroke survivors endure long-term disability, it is not surprising that the economic burden of stroke in the United States is projected to increase from $45.5 billion in 2014 to $129.3 billion by 2035 [79, 277]. Thus, increased efforts to understand, predict, and improve the functional outcome of stroke is essential.

Ischemic stroke (IS), which accounts for 87% of total stroke cases, carries a strong genetic basis with heritability estimates of 39% [22, 277]. The outcome of ischemic stroke is a multifactorial endpoint influenced by clinical and genetic variables [266, 277]. Most studies have implemented a candidate gene approach to examine associations between preselected polymorphisms and disability scores (see *Outcome metrics* below); however, many of the latest projects have been genome wide association studies (GWAS) attempting to validate prior findings and discover novel variants in a nonbiased fashion.

A comprehensive catalogue of known polymorphisms affecting poststroke recovery would serve as a roadmap for further studies, organizing relevant information for researchers to quickly grasp the state of the field and plan future directions. Furthermore, understanding the genetic underpinnings of ischemic stroke recovery can be leveraged for disability prediction, decision analysis, precision medicine, and drug target discovery. Understanding the polymorphisms and concomitantly affected molecular mechanisms is crucial to fully appreciate the impact on functional outcome. Here, we show that pertinent genes can be grouped by the following systems and processes: inflammation, vascular homeostasis, growth factors, metabolism, p53 regulatory pathway, and mitochondrial variation.

## Inflammation

### Cytochrome P450 pathway

**Fig. 1:**
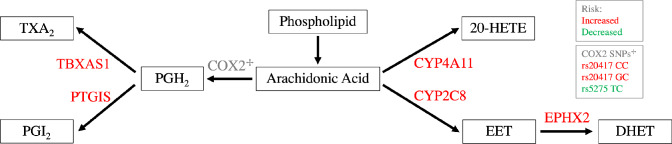
Depicts the eicosanoid inflammatory pathway. Genes shown in red contain a SNP associated with worse functional outcome. The COX2 locus contains both risk and protective variants which are detailed in the gray boxes. Abbreviations are as follows: thromboxane A2 (TXA2), prostacyclin (PGI2), prostaglandin H2 (PGH2), 20-hydroxyeicostetarenoic acid (20-HETE), epoxyeicosatrienoic acids (EET), and dihydroxyeicosatrienoic acid (DHET)

Cytochrome (CYP) P450s are a class of monooxygenase enzymes that, among other functions, metabolize arachidonic acid (AA) into eicosanoids such as 20-hydroxyeicostetarenoic acid (20-HETE) (Fig. 1). 20-HETE is a potent vasoconstrictor involved in the autoregulation of cerebral blood flow. It is associated with increased IS severity in animal models as well as larger lesions and worse modified Barthel Index (mBI) scores [69, 128, 286]. CYP epoxygenase enzymes also act on AA; however, they produce epoxyeicosatrienoic acids (EETs) that affect cerebral perfusion through vasodilation and offer protective effects against ischemia as opposed to 20-HETE [69, 128].

*CYP2C8 and CYP4A11*

The synthesis of 20-HETE is catalyzed by enzymes of the CYP4 gene family, while EETs are produced by CYP2C and CYP2J isoforms [128]. Interactions between CYP2C8, CYP4A11, and EPHX2 single nucleotide polymorphisms (SNPs) were associated with IS risk as well as increased 20-HETE and decreased EET [312]. One study in a Chinese population reported a higher frequency of CYP2C8 rs17110453 CC in patients with neurologic deterioration (ND) defined as a change in NIHSS score 10-days after admission. The same report revealed similar findings regarding CYP4A11 rs9333025 GG (Supplemental Table 1) [309].

*EPHX2*

Downstream in the CYP eicosanoid pathway, EETs are converted to dihydroxyeicosatrienoic acid (DHETs) by soluble epoxide hydrolases (sEH) (Fig. 1). DHETs are generally less bioactive than their precursor, thus sEH acts to reduce the vasodilatory and protective effects of EETs [69, 72, 128]. Accordingly, genetic variants in EPHX2, which encodes sEH, worsen IS outcomes. The missense rs751141 GG variant is independently associated with carotid stenosis [307], plaque density [314], increased metabolism of EET to DHET, and increased ND ten days poststroke leading to worse 3-month modified Rankin Scale (mRS) scores [308, 309].

### Cyclooxygenase pathway

*COX2*

Arachidonic acid is converted to prostaglandin H_2_ (PGH_2_) via cyclooxygenases encoded by COX-1 and COX-2. Various tissue-specific isomerases and synthases then generate PGH_2_ derivatives that trigger diverse biochemical cascades, examples of which include thromboxane A2 (TXA_2_) and prostacyclin (PGI_2_), synthesized by thromboxane synthase (TBXAS) and prostacyclin synthase (PTGIS), respectively (Fig. 1) [72]. With respect to cerebrovascular physiology, COX1 is implicated in the maintenance of vascular tone and vasodilator responses and evidence suggests that COX2 increases cerebral blood flow in accordance with neural activity [201, 202].

In addition to studies demonstrating that polymorphisms in COX genes affect IS risk, extensive investigations into how these changes affect functional outcome have also been conducted [326]. One group examined the effects of COX2 SNPs on a variety of functional outcome metrics including mRS, Glasgow Coma Scale (GCS), and BI at 90 days follow-up. Interestingly, they report an association between COX2 rs5275 TC and improved mRS, but not GCS or BI, while COX2 rs20417 GC was associated with better GCS scores but no other metrics [186]. Another group showed that COX-2 rs20417 CC was significant for ND [310] and a different investigation demonstrated that interactions between COX-2 rs20417, P2Y1 rs1371097, and GPIIIa rs2317676 were also associated with ND [311].

*TXAS1 and PTGIS*

The functional effects of SNPs in PTGIS and TBXAS1 were assessed alongside COX2 polymorphisms: TBXAS1 rs2267679 TT and PTGIS rs5602 CC occurred at a higher frequency in patients with ND. Furthermore, univariate analysis indicated that TBXAS1 rs41708 TT and PTGIS rs5629 CC were independently associated with ND [310].

### Toll-like receptors

Toll-like receptors (TLRs) are pattern recognition receptors that are an integral component of the innate immune system and can be activated by exogenous danger-associated molecular patterns (DAMPs) or endogenous ligands produced by disease and injury [140]. TLRs are interconnected with eicosanoid inflammatory pathways and can activate calcium-dependent phospholipase A2 that in turn, produce arachidonic acid-containing phospholipids [72]. Localized to microglial [165] and astrocytic [31] surfaces, TLR4 is implicated in neuroinflammation and stroke recovery [46, 292]. Specifically, increased TLR4 expression at 18–72 h and 7-days poststroke is associated with worse 90-day mRS scores [34, 272, 302]. Although many genetic variants exist in TLR4, two linked SNPs, *Asp299Gly* and *Thr399Ile,* occur at significant frequencies, forming the 299/399 haplotype [203]. One study examined the effects of 299/399 on IS functional outcome (ISFO) and found it to be predictive of worse 3-month mRS scores [288]. Proinflammatory markers such as IL-1β, I-L6, and TNFα are associated with TLR4 levels [34, 305] and elevated C-reactive protein 3-months poststroke is associated with 299/399 [288]. Thus, TLR perturbations likely affect other innate immune system processes.

### C-reactive protein

C-reactive protein (CRP) is not only an inflammatory biomarker but is also functionally active in compliment activation and Fc receptor binding, the latter of which stimulates cytokine release. Primarily synthesized in hepatocytes, transcriptional induction of CRP occurs in response to inflammatory cytokines including interleukin-6 (IL-6), interleukin-1 (IL-1) and tumor necrosis factor alpha (TNFα) [257]. Elevated CRP is a predictor of cardiovascular disease [67, 334], IS risk [328], and forecasts worse functional outcome (mRS) in acute IS patients according to multiple meta-analyses [127, 281]. Heritability estimates of baseline CRP levels range from 10 to 65% [233] and specific genetic variants affecting CRP levels have been studied in several disease contexts.

Many CRP SNPs have been described; however, only seven have been tested for their effects on ISFO. The minor allele of rs1130864, which is associated with elevated serum CRP in Han Chinese populations [149], demonstrated increased risk for poor outcome (3-month mRS) in the same ethnic group [108], It should be noted that rs1130864 has shown mixed associations with CRP levels in other populations [8, 9, 142, 148, 157], so caution is warranted when assuming the relationship shown in Guo et al. [108] will extrapolate to non-Asian cohorts. Two other SNPs, rs3093059 and rs11265260, were identified as independent risk factors for elevated CRP and worse 3-month mRS scores in Han Chinese patients. In addition, a haplotype analysis of five SNPs including the two previously mentioned was also included in the study and showed significance for poor outcome [306].

### Cytokines

*IL-1 and IL1RN*

Interleukin-1 is an archetypal cytokine involved in a myriad of proinflammatory events that occur during acute brain injury. Pertinent examples include matrix metalloproteinase and platelet activation, augmented angiogenesis, diminished neurogenesis, and the induction of other cytokines [251]. IL-1⍺ and IL-1β are well-studied agonists of the IL-1 type I receptor (IL-1R1), while IL-1 receptor antagonist (IL-1Ra) is a potent endogenous inhibitor [246]. Preclinical studies investigating the therapeutic potential of IL-1Ra administration yielded promising results, yet in clinical trials, the use of IL-1Ra reduced inflammatory biomarkers but failed to show a clear association with improved functional outcome [250, 251]. IL1RN encodes IL-1Ra and contains a variable number tandem repeat polymorphism in which the minor allele (*2) increases IL-1Ra production [68]. Homozygotes (IL-1RN 2/2) had improved clinical outcomes (BI; 7-days, 1-month, 3-months, 1-year) in an IS study [105] and in Rezk et al. which had a cohort composed of patients with intracranial hemorrhage, subarachnoid hemorrhage, and IS [223]. Rezk et al. also reported that a biallelic variant in the promotor region of IL-1β (-511C/T; rs16944), known to increase secretion [217], was associated with worse BI scores at various timepoints out to 6-months poststroke. They demonstrated similar findings for an intronic polymorphism of IL-1⍺ (-889C/T) [223].

*IL-6*

Interleukin-6 is a pleiotropic cytokine capable of complex, context-dependent functionality [84, 154]. Regarding IS, the exact of role IL-6 remains nebulous. In vivo studies have shown through a variety of approaches including gene knockout [96], intracerebroventricular administration [182, 194], and receptor blockage [299] that IL-6 is neuroprotective [49]. Nonetheless, a plethora of evidence suggests IL-6 is elevated in stroke and is significantly correlated with lesion size [249, 263] and worse functional outcome [12, 42, 237].

A variant in the IL-6 promoter (− 174 G/C; rs1800795) is known to influence IL-6 levels; however, controversy exists regarding which allele increases IL-6 expression and IS risk [152, 268]. One group reported the GG genotype as protective: it was associated with decreased serum IL-6 levels as well as improved 7-day (NIHSS), 3-month (mRS, BI), and 6-month (mRS, BI) outcomes in an Indian population [50]. Contrarily, another study conducted in young patients yielded the opposite findings: the GG genotype was a risk factor for elevated RS at 7-days and 3-months poststroke [102]. The most recent investigation was inconclusive, finding no association between rs1800795 and poststroke IL-6 levels or 6-month mBI [301]. These inconsistencies may be attributed to population differences or experimental biases. Further investigation is warranted to properly elucidate the effects of rs1800795 on ISFO. IL-6 receptor polymorphism Asp358Ala (+ 48,892 A/C; rs8192284) which increases IL-6 levels [337] was shown to improve 3-month mRS scores suggesting that increased IL-6 levels is indeed therapeutic in poststroke recovery [130].

*IL-10*

Interleukin-10 is generally considered an anti-inflammatory cytokine and is expressed in response to brain injury. It acts by limiting proinflammatory cytokines and effector actions of T cells, monocytes, and macrophages [94]. Preclinical evidence suggest that IL-10 is neuroprotective and reduces infarct volume [24, 26, 169, 172, 256]. With some exceptions [51, 200], clinical studies indicate that low IL-10 levels accompany IS [87, 126, 248] or coincide with acute neurological deterioration [220, 276]. One relevant IL-10 gene promoter polymorphism (–1082 G/A; rs1800896) has mixed evidence regarding its influence on ischemic stroke risk [153, 315]; however, one group did find a significant association between rs1800896 GG and lower BI scores at 1-month and 3-months poststroke [192]. Other SNPs increase IS risk (rs1800872, rs1554286, rs3021094) [297], but remain untested in the context of functional outcome [155].

*RETN*

Resistin is a pro-inflammatory, atherogenic adipokine associated with acute cerebral infarction independent of obesity-related pathways [206]. Elevated serum resistin was linked to 5-year mortality and poststroke disability (mRS) [82, 163]. The -420 C/G (rs1862513) polymorphism in the promoter region of RETN leads to increased resistin levels [58, 162, 207]. Bouziana et al. reported that carriers of the minor allele present with more severe IS and experience higher in-hospital mortality [30]. Interestingly, the same group also found an association between -420G and improved 1-year mRS scores. The authors postulate the contradiction stems from selection bias whereby -420G carriers who endure increased risk of acute death may create artificial associations with long-term recovery [29].

*circ-STAT3*

Noncoding, circular RNAs (circRNAs) are ubiquitous in neural tissue and differentially expressed in stroke patients with subtype-specific profiles [78, 178, 208]. One group was able to predict IS outcomes by measuring only three circRNAs, thus demonstrating robust biomarker potential [333]. A growing number of associations between circRNA variants and human disease includes connections to atherosclerosis [41], multiple sclerosis [210], and coronary artery disease [330]. The only study investigating functional recovery reported that circ-STAT3 rs2293152 GG worsened poststroke disability (3-month mRS) [180]. Signal transducer and activator of transcription 3 (STAT3) molecules are regulated by Janus kinases 2 (JAK2). JAK2/STAT3 activation exacerbates neuroinflammation and inhibition of this pathway can mitigate cerebral ischemic injury and decrease infarct size after stroke [331]. Liu et al. posits that rs2293152 GG influences STAT3 levels by altering circ-STAT3 expression, or by modifying its ability to bind regulatory miRNAs [180].

### Myeloperoxidase

Myeloperoxidase (MPO) is a heme-containing enzyme found in primary azurophilic granules of neutrophils. MPO augments innate immune system defense via production of reactive oxygen species (ROS) such as hypochlorous acid [11]. Considering that exuberant ROS production leads to host tissue damage, and that MPO levels increase poststroke [61], MPO inhibition as a therapeutic strategy has been posed and successfully tested in animal models [146]. A candidate gene approach was employed to examine the effects of MPO polymorphisms on ISFO and G463A (rs2333227) was associated with worse RS scores at follow-up (median interval: 11 days) [125]. Another variant, rs2107545 CC, led to poor 6-month outcomes (mRS, BI) [175].

## Vascular homeostasis

### Hemostasis

*GPIIIa*

Normal platelet functioning is essential for primary hemostasis and thrombosis [214]. Elevated platelet markers in early cerebrovascular studies led authors to postulate that increased platelet activity may be pathogenic, at least in certain stroke subtypes [238, 270, 271]. This notion meshes well with the contemporary practice of antiplatelet administration for treatment and prophylaxis of ischemic events. Platelet adhesion and aggregation is orchestrated in part by integrins, a class of glycoprotein signaling receptors [129]. The constellation of integrin species on human platelets includes α_IIb_β_3._ This transmembrane protein offers binding sites for hemostasis-related ligands such as Von Willebrand factor, fibrinogen, fibronectin, and CD40 [229]. Experiments in nonhuman primate models showed microvascular preservation after α_IIb_β_3_ inhibition, so genetic alterations to GPIIa and GPIIIb which encode the α_IIb_ and β_3_ subunits may affect stroke outcome [5, 47, 129].

While multiple groups have reported that genetic variants in GPIIb/GPIIIa affect IS risk, evidence of their influence on functional outcome is less robust [176]. Initial investigations revealed that poststroke mortality is affected by the HPA-3 genotype of GPIIb with 80% of *bb* carriers surviving at follow-up, compared to 68% of *ab,* and 58% of *aa* carriers [47]. Another study showed that the A2 allele of GPIIIa rs5918 was significantly associated with poor BI scores at 90-days postadmission, but not mRS or GCS grades [186]. As previously mentioned, interactions between GPIIIa rs2317676, COX-2 rs20417, and P2Y1 rs1371097 were significant for ND ten days after admission [311]. P2Y1 is a G protein-coupled receptor classically known to activate platelet aggregation, but also participates in neuroinflammatory processes via IL-6 induction [92, 316]. These molecules certainly contribute to stroke pathophysiology considering that P2Y1 receptors are upregulated during cerebral ischemia and infarct size is reduced after P2 receptor antagonism [159, 269].

*⍺2AP*

Alpha-2-antiplasmin (⍺2AP) influences fibrinolysis via plasmin deactivation [1]. One ⍺2AP mutation, Arg407Lys (rs1057335), is associated with cardiovascular disease as demonstrated in Bridge et al. where carriers of 407Lys were at decreased risk for abdominal aortic aneurysm [35]. Relating to cerebral ischemia, a multivariate analysis indicated that IS and transient ischemic attack (TIA) patients with the minor allele were more likely to have long-term functional recovery (mRS, 6–12 months). These findings suggest a protective role for Arg407Lys, but the exact mechanisms underlying this effect remain obscure [296].

*eNOS*

**Fig. 2:**
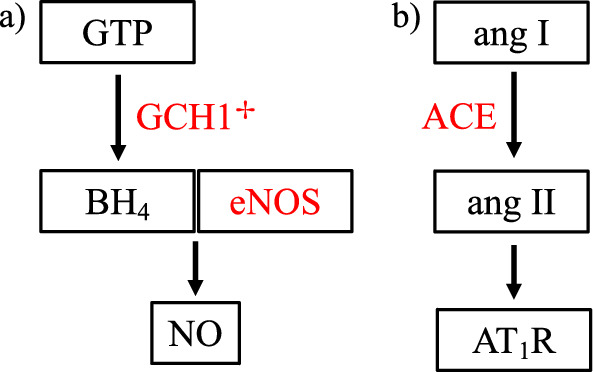
has two components: **a** and **b**. **a** Shows the molecular pathway for nitric oxide (NO) synthesis and shows that genetic variants in GCH1 and eNOS lead to adverse outcomes after stroke. ✢ placed to clarify that the GCH1 variant led to death after IS but was not associated with a functional outcome metric. **b** Shows pathway for angiotensin II type 1 receptor (AT_1_R) agonism which is augmented by an angiotensin converting enzyme (ACE) polymorphism and leads to worse poststroke recovery

Nitic oxide (NO) is a lilliputian signaling molecule and orthodox vasodilator with anti-proliferative and anti-thrombotic properties [88]. NO also facilitates synaptic plasticity and is neuroprotective at physiologic concentrations yet neurotoxic at higher levels [44]. Three isoforms of nitric oxide synthase (NOS) produce NO including endothelial (eNOS), neuronal, and inducible versions. NOS requires several cofactors including tetrahydrobiopterin (BH_4_), the synthesis of which is governed by rate-limiting enzyme, GTP cyclohydrolase 1 (GCH1) [90]. Polymorphisms affecting eNOS and GCH1 have been linked to adverse poststroke outcomes (Fig. 2a). For example, G894T (rs1799983) is a missense variant altering the active site of eNOS. Asian carriers of the T allele endure increased risk of IS, unlike Caucasians [56, 151]. However, one study found that Mediterranean patients with the mutation have worse functional outcomes (1-month mRS) [83]. Regarding GCH1, + 243C/T is a SNP in the 3'-UTR that decreases NO production leading to increased vascular events and death after IS (5-year follow-up) [262, 319].

### Atherosclerosis

*ACE*

The renin angiotensin system (RAS) is integral to vascular homeostasis and is also active in brain parenchyma, influencing learning and memory [65]. In the classical RAS axis, angiotensin converting enzyme (ACE) cleaves angiotensin I to produce the angiotensin II (ang II) (Fig. 2b). Overactivation of ang II type 1 receptor (AT_1_R) is implicated in stroke pathogenesis via proinflammatory, profibrotic, and vasoconstrictive effects. Increased oxidative stress also contributes to these proatherosclerotic changes [14]. ACE is a key player in aberrant RAS activity as evidenced by studies wherein pre-stroke ACE inhibition reduced ischemic stroke incidence [120] and severity [57], and improved poststroke recovery (BI at discharge) [116]. A 287-bp insertion/deletion (I/D) polymorphism within the ACE gene is known to affect serum ACE levels. Carriers of the D allele possess higher levels of ACE [224], incur increased risk of IS [323], and are subject to worse functional outcomes (BI at discharge) [189].

*HDAC9*

Histone deacetylases (HDACs) remove acetyl groups from lysine residues on histones and other regulatory proteins, promoting a heterochromatic configuration that dampens transcriptional activity [110]. HDAC inhibition is a common therapeutic approach and has shown promise in preclinical stroke models [160]. Several variants at the HDAC9 locus are associated with IS risk [111, 164, 267, 335]. Two of these variants, rs2074633 and rs28688791, are associated with unfavorable short-term outcome (3-month mRS) and are in linkage disequilibrium with another risk SNP, rs2107595 [282]. This variant is associated with common carotid intimal thickness, the presence of carotid plaque, and increased HDAC9 expression [17, 191]. Mechanistic studies found that HDAC9 represses cholesterol efflux and promotes atherosclerosis [45].

*TNFRSF11B*

Osteoprotegerin (OPG) is a member of the tumor necrosis receptor superfamily with roles in bone homeostasis, vascular inflammation, and calcification [274]. Whether OPG activity is beneficial or harmful in the context of vascular disease remains unclear. While OPG knockout accelerates calcific atherosclerosis in certain animal models [40], suggesting protective effects, preclinical stroke models showed reduced infarct volume and brain edema in OPG^−/−^ mice, indicating a pathogenic role for OPG in cerebrovascular disease [240]. Clinically, OPG levels positively correlate with stroke severity [46], mortality [132], and poor functional outcome (3-month mRS) [254]. Two genetic variants in TNFRSF11B, which encodes OPG, are associated with increased serum OPG levels [260], IS occurrence [25], and worse poststroke recovery (rs2073617G, rs3134069C; 3-month mRS) [283]. In silico analysis projected that these SNPs are in histone modification regions, hence the altered OPG levels [283].

*PDE4D*

Cyclic adenosine monophosphate (cAMP) is a ubiquitous second messenger generated by adenylyl cyclase that facilitates diverse cellular processes in response to various stimuli. The breakdown of cAMP to inactive AMP is catalyzed by phosphodiesterase (PDE) enzymes [234]. A connection between stroke and PDEs was discovered when Gretarsdottir et al. reported that certain PDE4D haplotypes were associated with IS susceptibility. The authors posited atherosclerosis-related mechanisms as an explanation considering the strongest associations were among cardiogenic and carotid stroke subtypes [103]. A decade of additional studies summarized by meta-analysis confirmed that some PDE4D variants are indeed related to stroke [181]. And while cAMP does inhibit a variety of proatherosclerotic changes in vascular smooth muscles cells including proliferation [131], migration [95], and collagen deposition [81], it also mitigates neuroinflammation and increases BDNF expression [298]. PDE4D variant, SNP87 (rs2910829), although not related to IS incidence overall [170], was found to worsen functional outcomes (3-month mRS) [255].

*OPN*

Osteopontin (OPN) is a multifunctional phosphoglycoprotein that guides numerous signaling pathways and in the context of acute brain injury, balancing pro-inflammatory and anti-inflammatory responses to modulate neuroinflammation, apoptosis, and blood–brain barrier (BBB) integrity [329]. Some evidence also suggests that OPN potentiates atherosclerosis [294]. Nonetheless, several in vitro studies have indicated a neuroprotective role for OPN; specifically, OPN administration reduced infarct volume and lessened neurologic deficits in rodent models of IS [134]. Clinically, however, elevated OPN is associated with worse outcomes (3-month mRS [195], recurrence [93]). This correlation may be a reactive phenomenon in which compensatory increases in OPN occur in patients with more severe stroke. OPN polymorphism -443 C/T (rs11730582) was examined in IS patients and investigators reported that -443 CC was associated with decreased levels of a particular OPN isotope and that CC homozygotes experienced worse long-term functional recovery (12-month mRS and BI) reinforcing the notion that OPN is neuroprotective [135].

## Growth factors

*BDNF*

Brain-derived neurotrophic factor (BDNF) is a highly expressed protein involved in numerous nervous system activities including neuroinflammation, neuronal survival, and plasticity [19]. BDNF administration in stroke animal models has demonstrated various benefits including reduction of infarct size [235] and improved functional outcome [198]. In humans, BDNF levels are negatively correlated with NIHSS scores [139] and low initial quantities are associated with worse mRS outcomes at 90-days [161, 280], 2-years, and 7-years poststroke [258].

BDNF is initially synthesized as a precursor protein (proBDNF) that is cleaved to separate a mature domain (mBDNF) and prodomain. All three molecules are bioactive and exhibit distinct, even opposing effects due to differing receptor affinity [71]. Among multiple SNPs in the BDNF gene, val66met (rs6252; G196A), which occurs in the prodomain, is the most studied variant in the context of IS. Molecularly, val66met reduces activity-dependent secretion of BDNF [18].

Val66met is implicated in the pathogenesis of many neuropsychiatric disorders [123] and multiple groups have reported associations with IS occurrence [141, 325, 327]. In aneurysmal subarachnoid hemorrhage, met66 is a negative prognostic factor [244]. Several studies have investigated the effects of val66met on ISFO, some of which are aggregated in a meta-analysis that reports an overall significant association between the met allele and unfavorable outcome as defined by study-dependent mRS cutoff values [193]. Nonetheless, other publications utilizing mRS as an outcome metric have yielded mixed results with some groups reporting negative findings [141]. Recently, two studies of large cohorts reported contrasting findings: Braun et al. found that poor outcome (mRS ≥ 3) was associated with met66 (n = 829) in a young American population (average age = 41.4) [33], while Zhou et al. described no such association in a Han Chinese population (mRS ≥ 2; n = 778; average age = 64) [327]. Differences in study parameters and population demographics may account for the incongruity.

Aside from mRS, other outcome measurements have bolstered the case for val66met as a risk factor for IS recovery. BI at 6-weeks postrehabilitation [232] and cognitive functional independence Measure (FIM) at discharge [112] are more likely to be compromised in patients carrying the met66 allele. In addition, more granular phenotypes have been studied as well. Specifically, val66met is associated with decreased motor function [52] and improvement [242, 278], rate of adaptation [122], and sensorimotor cortex activation after stroke [145]. In patients with dysphagia, the met66 allele was originally described as protective [85]; however, new evidence suggests the opposite [204]. Other BDNF SNPs, although less studied than val66met*,* have also been linked to poststroke disability. For example, rs11030119 is an intronic variant associated with favorable 7-year mRS [259]. And rs7124442, which is located the 3'-UTR and affects miR-922 binding, is significant for increased BDNF expression and improved NIHSS scores at 3-months poststroke [174].

*IGF1*

Insulin-like growth factor 1 (IGF1) is a polypeptide hormone that activates signaling pathways to promote growth, neurodevelopment, and neuroplasticity [295]. IGF1 decreases with age [138], but remains an important neuroprotective agent as evidenced by clinical studies that, minus one exception [13], show elevated serum IGF1 levels correlating with improved functional outcomes in stroke patients [2, 70]. In vivo stroke models also support this notion: IGF1 administration reduces infarct volume [236]. Regarding IGF1 genetics, multiple SNPs have been identified in addition to a 192 base pair CA repeat polymorphism in the promoter region that increases serum IGF1 concentrations [273] and decreases IS risk [225]. With respect to functional outcome, one study demonstrated that rs7136446 was significantly associated with lower IGF1 levels in healthy controls and worse mRS scores 2-years poststroke [3].

*VEGF*

Vascular endothelial growth factor (VEGF) is a signaling protein that stimulates angiogenesis and is neuroprotective as evidenced by preclinical models wherein VEGF administration reduces infarct volume and cognitive deficits after ischemic events [101]. Polymorphisms in VEGFA have been linked to cardiovascular disease including stroke [147, 285]. Regarding functional outcome, one group reported that + 936C/T (rs3025039) led to worse IS outcomes (3-month mRS) and that this variant is in linkage disequilibrium with nearby SNP, + 1451C*/*T (rs3025040), both of which occupy the 3'-UTR. Subsequent experimentation revealed that + 1451C*/*T lies within a crucial binding cite for regulatory miRNAs (miR-199a, miR-199b) with the minor allele decreasing miRNA binding affinity and reducing VEGFA expression [324]. A variant in the VEGFA receptor, VEGFR2 (+ 1719A/T; rs1870377T/A; Q472H), elevates serum VEGF [245], increases microvessel density [97], and improves 3-month mRS scores [168].

*ANGPT1*

While VEGF promotes early angiogenesis, Angiopoietin 1 (Ang1) acts later by decreasing vascular permeability and inflammation, leading to BBB preservation and periinfarct neovascularization after ischemic injury in preclinical studies [113, 265]. Accordingly, ischemic stroke patients with low plasma Ang1 levels at admission had worse 3-month mRS scores [98]. Two SNPs in the ANGPT1 3'-UTR reportedly alter IS risk, one of which also worsened poststroke recovery in a Chinese population (rs2507799; 1-month mRS) [53, 54]. Differential binding of miR-607 potentially explains why carriers of the T allele had decreased plasma Ang1 and poorer outcomes [53].

## Metabolism

*APOE*

Apolipoprotein E (APOE) is a ubiquitously expressed glycoprotein found in a variety of central nervous system cell types, facilitating the transport and metabolism of cholesterol and other lipids [300]. After being secreted from cells, APOE becomes lipid-bound and carries cargo molecules to neuronal cell-surface APOE receptors such as low-density lipoprotein receptor (LDLR). Polymorphisms in two critical positions on the APOE gene generate three allelic variants: ε2, ε3, and ε4. APOE ε2 (APOE2) binding to LDLR is 50 times weaker than the other alleles, while APOE4 has enhanced binding to very low-density lipoprotein (VLDL) particles [300]. Consequently, pathologies are dichotomized by deficient or excessive lipoprotein processing. In the periphery, APOE2 is associated with Type-III hyperlipoproteinemia, and APOE4 with proatherogenic lipoprotein conditions [216, 300]. In brain parenchyma, APOE is involved in cerebrovascular function, glucose metabolism, and synaptic integrity and plasticity although the exact mechanisms underlying these relationships are poorly understood [300].

APOE4 genotype is a major risk factor for Alzheimer’s disease (AD), increasing Aβ plaque formation [219]. Contrarily, APOE2 is protective for AD and associated with reduced plaque burden [64]. APOE impacts cerebrovascular disease as well: cerebral amyloid angiopathy (CAA), which is modulated by APOE4 [222], increases risk for lobar intracranial hemorrhage (ICH) [230], the severity of which is impacted by APOE2 [39]. Although APOE influences IS risk [143], its effect on poststroke disability remains controversial.

Several studies have examined the relationship between APOE4 genotype and ISFO. A recent meta-analysis of relevant publications from 1998 to 2012 reported no overall statistically significant association [193]. This analysis included measurements from a variety of timepoints (1-month, 3-months, 1-year), quantified by various metrics (NIHSS, BI, RS, mRS, FIM, mortality). Another group reported negative findings regarding functional outcome, but found that males with the APOE4 allele endured higher 1-year mortality, poststroke [104].

Further negative findings outside of those encompassed in the meta-analysis include additional studies reporting no association between APOE4 and RS [185], or APOE4 and mRS [158, 289, 321]. Interestingly, APOE4 decreases age of ischemic stroke onset [158], and specific cognitive testing has shown that APOE4 delays the recovery of verbal memory functioning 1-year poststroke [289]. In addition, ε4 homozygosity leads to poststroke dementia [213]. Nonetheless, underwhelming evidence exists regarding the effects of APOE4 and ISFO.

APOE2 is less studied in the context of stroke outcome. Broderick et al. found that ε2 carriers treated with t-PA had better 3-month outcomes (mRS) [36]; however, a general effect on poststroke recovery has not been shown [321], only a sex-specific association whereby male carriers of the ε2 allele experienced increased disability (3-month mRS) [158].

*MTHFR*

**Fig. 3:**
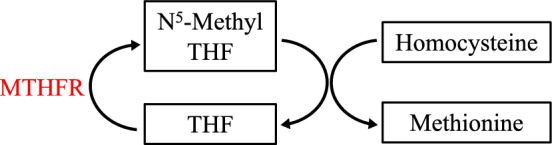
Depicts methionine regeneration and its dependence on methylene tetrahydrofolate reductase (MTHFR), a genetic variant of which leads to worse functional outcomes after stroke. THF is abbreviation of tetrahydrofolate

Homocysteine is a member of the sulfur-containing amino acid metabolic pathway. Methylene tetrahydrofolate reductase (MTHFR) regenerates N^5^-methyl-tetrahydrofolate, so homocysteine can be used to replenish methionine, an essential amino acid (Fig. 3) [38]. Alterations to MTHFR efficiency can cause homocysteine buildup resulting in a multitude of pathologic effects including endothelial dysfunction, ROS generation, DNA repair suppression, and increased apoptosis [166]. Two well-studied variants, C667T and A1298C, reduce enzyme activity by 75% and 39%, respectively in homozygous carriers [279]. Both mutations increase ischemic stroke risk [4, 77] but have no known associations with poststroke recovery. He et al. examined rs868014, a SNP in the 3'-UTR, and reported increased miR-1203 binding leading to decreased MTHFR expression, heightened serum homocysteine, increased stroke incidence and severity, as well as worse functional outcomes (3-month ΔNIHSS). Interestingly, rs868014 was linked to A1298C [118].

*COMT*

Catechol-O-methyltransferase (COMT) metabolizes dopamine and thus inhibits its activity in the synaptic cleft [291]. Dopamine, which massively increases during ischemic stroke onset, influences motor activity, memory, and cognition among other functions. While dopamine-enhancing drugs almost universally improve sensorimotor function in a variety of animal stroke models, clinical trials have failed to reproduce the same success: dopamine augmentation failed to improve general disability tests such as BI in most trials and showed mixed results when motor-specific scales were used as outcome variables [100]. COMT contains a key polymorphism, val158met (rs4680), that decreases enzymatic activity, increasing dopamine in the synaptic cleft by as much as 38% [291]. This variant significantly worsens BI and Rivermead Motor Assessment (RMA) at 1-month and 6-months poststroke as well as FIM and Fugl-Meyer Assessment (FMA) at 3-months and 6-months poststroke [144, 171]. These findings suggest a harmful role of excess dopamine in IS recovery that contradicts preclinical models.

*NOX4*

NADPH oxidase (NOX) enzymes generate ROS that propagate redox dependent signaling pathways to execute numerous physiologic functions [275]. ROS abundance is an essential component of stroke pathophysiology causing lipid peroxidation, DNA damage, and apoptosis [205]. NOX4 is induced under hypoxic conditions and is especially harmful during cerebral ischemia [48]. NOX4 variant rs11018628 TC/CC is associated with decreased IS risk and improved functional recovery (ΔNIHSS at discharge) [119].

### Hypothalamic-pituitary-thyroid axis

Tightly controlled by a precise feedback loop known as the hypothalamic-pituitary-thyroid (HPT) axis, thyroid hormones affect metabolism, development, and growth, influencing virtually every cell in the human body. The HPT axis is as follows: thyrotropin-releasing hormone (TSH) generated in the hypothalamus causes thyroid-stimulating hormone (TSH) secretion in the hypophysis, precipitating T3 and T4 synthesis in the thyroid gland at a 1:14 ratio. T4 is converted to the more biologically active T3 by deiodinase enzymes in extrathyroidal tissues [184]. Many studies investigating interplay between thyroid hormones and stroke outcome have been conducted. One meta-analysis revealed an overall association between poor mRS scores and low T3 levels [133]. Decreased T3 has also been associated with increased 1-year mortality [7] and worse NIHSS scores (~ 2–4 weeks) [322]. Thus, T3 appears to be neuroprotective in acute cerebrovascular disease. Regarding other HPT hormones, high T4 is prognostic for long-term disability [133] and although individual studies report mixed findings regarding the effects of TSH on ISFO, a meta-analysis found that initially elevated TSH levels improved 1-month and 3-month mRS scores [74].

*DIO3*

Given the impact thyroid hormones have on IS outcome, genetic variants affecting their transport and activity were postulated to influence poststroke disability. Deiodinase 3 (DIO3) is an enzyme that inactivates T3 in neurons—a process induced by hypoxic conditions [136]. One group found that DIO3 SNP rs945006 TT was associated with improved ISFO at 1-year follow up (mRS) [264]. The mechanisms by which rs945006 affects intraneural DIO3 activity are currently unknown.

*OATP1C1*

Organic anion transporting polypeptide 1C1 (OATP1C1) is a transmembrane protein specific for thyroid hormone transport in the brain and animal models indicate that inflammation reduces OATP1C1 mRNA expression in cerebral blood vessels [21, 293]. One group reported a relationship between rs10770704 CC and increased disability 1-year poststroke (mRS) [264], despite multiple studies reporting no direct effect of the SNP on thyroid hormone levels [73, 227].

## p53 regulatory pathway

*TP53 and MDM2*

**Fig. 4:**
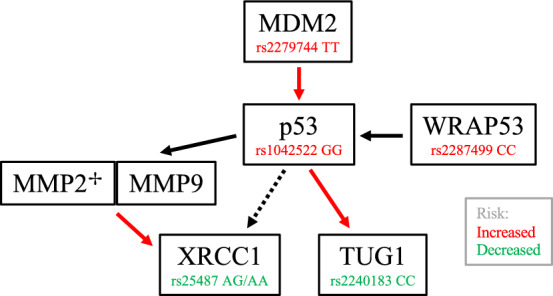
illustrates the various components of the p53 regulatory pathway and relevant polymorphisms associated with ISFO. Red arrows indicate negative regulation and the dotted arrow from p53 to XRCC1 denotes the indirect relationship between the two. ✢: as noted above, numerous MMP2 SNPs were found but did not endure Bonferroni correction

Neuronal apoptosis is a determinate of penumbra progression and highly influenced by the p53 regulatory pathway. A variety of stress signals including hypoxia and DNA damage can trigger p53 activation leading to cellular death, while p53 attenuation is therapeutic in animal models of IS [124]. Genetic variants affecting p53 and its regulators and downstream targets have been implicated in poststroke recovery. The *arg72pro* (rs1042522) SNP of TP53, which encodes p53, alters its proapoptotic functionality. *Arg72* augments apoptosis, a seemingly hazardous modification for IS patients considering the *arg/arg* genotype worsens poststroke disability (3-month mRS) [99]. Murine double minute 2 (MDM2) is an E3 ubiquitin protein ligase and direct negative regulator of p53 (Fig. 4). The minor allele of MDM2 SNP309 (rs2279744) increases MDM2 expression, downregulates p53, and improves 3- and 12-month mRS scores [226] at the cost of increased tumor formation [27].

*TUG1*

Taurine-upregulated gene 1 (TUG1) is a long non-coding RNA induced by p53 and upregulated in IS (Fig. 4). In vitro studies indicate that TUG1 knockdown reduces neuronal apoptosis in oxygen–glucose deprivation (OGD) models, while decreased TUG1 expression is associated with worse cancer outcome metrics [55, 317]. One group showed that the C allele of rs2240183, a SNP in the TUG1 promoter region, led to increased TUG1 expression and IS risk which is in align with the aforementioned preclinical evidence [287]. However, Liu et al. reported the CC genotype as protective with respect to 3-month mRS scores [179], thus further investigation is required to confirm the effects of rs2240183 on TUG1 activity and resultant changes in IS risk and outcome.

*WRAP53 and XRCC1*

Ischemia–reperfusion injuries after stroke generate ROS leading to DNA damage [167]. WD40-encoding p53-antisense RNA (WRAP53) is a regulatory RNA that facilitates p53-mediated DNA repair and apoptosis (Fig. 4) [188]. Relating to stroke, it has been shown that WRAP53 is translocated to the nucleus upon hypoxic injury where it is essential for the assembly of DNA repair-related proteins and neuronal survival. A coding variant of WRAP53 (rs2287499; *arg68gly*) increases the rate of nuclear translocation and thus enhances repair. Homozygous carriers of the less efficient *arg68* allele have larger infarct volumes and worse functional outcomes (3-month mRS) after IS [231]. X-ray repair cross-complementing protein 1 (XRCC1) is a scaffold protein for DNA repair enzymes integral to the DNA single strand break repair pathway [75], and in the context of base excision repair, is indirectly regulated by p53 (Fig. 4) [218]. In functional outcome studies, XRCC1 SNP rs25487 was shown to reduce IS susceptibility and improve short-term recovery (ΔNIHSS from admission to discharge) [117].

*MMP2*

Matrix metalloproteinases are proteolytic enzymes that cleave extracellular matrix proteins to aid in cell motility, tissue remodeling, and inflammatory responses [209]. In stroke, acute hypoxemia triggers MMP2 release from astrocytes and subsequent degradation of BBB integrity. If reperfusion is not accomplished, free radicals induce MMP9 production in microglia and pericytes further damaging the cerebrovasculature [304]. Accordingly, MMP inhibition abrogates BBB insult [228] and reduces apoptosis [107] in preclinical models. In addition, MMP2 and MMP9 exhibit intranuclear activity and can degrade DNA repair proteins including XRCC1, worsening DNA damage after ischemic events (Fig. 4) [303]. Evidence suggests that p53 regulates both MMP2 and MMP9 expression [23, 196]. Regarding ISFO, one study reported significant associations between nine MMP2 variants (Supplemental Table 5) and 3-month mRS scores after leveraging linkage disequilibrium parameters. These associations did not persist after Bonferroni correction [190]. Although no associations with MMP9 were discovered individually, gene–gene interactions between TP53 rs1042522, MDM2 rs2279744, and MMP9 rs3918242 were significant for ND (10-day NIHSS increase ≥ 2) and increased poststroke disability (3-month mRS) [313].

## Mitochondrial variation

*Haplogroups and UCP2*

Mitochondria enable oxidative phosphorylation and are intimately involved in apoptosis, both of which are key processes affecting cell survival under ischemic conditions [247]. As a result, perturbations of the mitochondrial genome or nuclear genes encoding mitochondrial proteins impact poststroke recovery. Specifically, haplogroups N9 [43] and R0 [66] are protective for ND (14-day and 1-month ΔNIHSS, respectively), but are otherwise unstudied in the context of stroke. The association with N9 was identified in an pure IS cohort [43], while Cramer et al. discovered the R0 association in a population composed of 77% ischemic stroke patients [66]. With respect to nuclear DNA, one group found a highly protective variant (-866G/A, rs659366) in the UCP2 gene which encodes uncoupling protein 2, localized to the inner mitochondrial membrane. After recanalization, patients with the AA genotype were twenty times more likely to be functionally independent (3-month mRS). Preclinical studies suggest the uncoupling action of UCP2 reduces ROS generation and the A allele of -866G/A increases UCP2 expression [32, 76, 86].

*mtDNA-CN*

Mitochondrial DNA copy number (mtDNA-CN) is a proxy for mitochondrial function and has been linked to cardiovascular disease, neurodegeneration, and aging [183]. Low mtDNA-CN is associated with increased stroke incidence, severity, disability (1- and 3-month mRS), and mortality [16, 59, 253]. While the exact mechanism underlying these associations remains unclear, a role for mtDNA-CN in BBB preservation and inflammatory modulation has been postulated [59].

## Genome-wide association studies

*PATJ*

As the name suggests, PALS1-associated tight junction protein (PATJ) is a macromolecule involved in tight junction formation and epithelial cell polarity [241]. PATJ is also implicated in the PI3K-Akt signaling pathway [20]. Previously unrelated to stroke, a recent meta-analysis of genome-wide association studies (GWAS) found 18 low-frequency intronic SNPs in the PATJ locus to be significantly associated with 3-month mRS scores. An additive model suggested that every G allele of the lead variant, rs76221407, led to a 0.4-point increase in mRS score [197]. It has been hypothesized that the mechanisms through which PATJ perturbations affect poststroke disability are related to altered angiogenesis, axonal regeneration, and BBB continuity [284].

*LOC105372028*

OC105372028 is an understudied gene of unknown biological significance. Söderholm et al., conducted a meta-analysis of GWAS data and established a connection between rs1842681, a variant at the locus, and ISFO (3-month mRS) albeit with a small effect size (OR = 1.12–1.40) [252]. The authors speculate that LOC105372028 affects brain plasticity through regulatory actions on PPP1R21 which in turn modulate protein phosphatase 1 (PP1) levels. PP1 is involved in many nervous system behaviors including learning and memory [199].

*PTCH1*

Söderholm et al. also reported suggestive findings regarding PTCH1 SNP, rs2236406 [252]. Although statistical significance wasn’t achieved, another group employed a candidate gene approach in a Chinese population to confirm its association with 3-month mRS [332]. PTCH1 is a cell surface receptor that initiates Sonic hedgehog (Shh) signaling which is canonically related to cellular differentiation and organogenesis, but also implicated in poststroke neurogenesis and recovery [320].

*Structural variation*

**Fig. 5:**
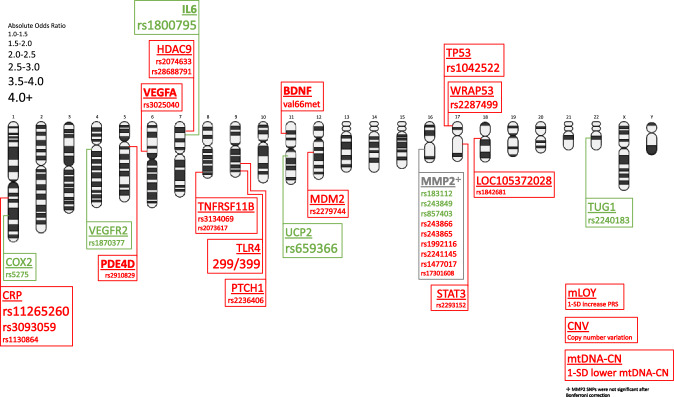
Maps genetic variants associated with 90-day mRS to a chromosomal ideogram. If the locus is bold, then the threshold for favorable outcome is mRS < 2, otherwise, the cutoff is mRS < 3. Red text indicates risk variants, while green text signifies protective variants. The font size of loci names is unchanging; however, the font size of a particular variant at that locus scales with absolute odds ratio. Absolute odds ratio permits protective and risk variants to be scaled together. For example, UCP2 rs659366 lessens likelihood of poor functional outcome with an odds ratio of 0.05, or absolute odds ratio of 20, therefore, it is given the largest font size and is colored green to indicate a large protective effect

Genome wide approaches have also highlighted the impact of genetic imbalance on stroke outcome. Copy number variations (CNV) are large scale genomic alterations that can occur as benign polymorphisms or drive pathology in human disease [318]. CNV has been extensively studied in the context of vascular disease and is associated with increased stroke risk as well as certain stroke subtypes [106]. CNV of dose-sensitive genes independently contributed to unfavorable outcomes in IS patients (3-month mRS) [215]. Future studies aim to further elucidate the impact of CNV on stroke risk and outcome [62]. Mosaic loss of chromosome Y (mLOY) is an insidious progression of aneuploidy in somatic cells. Traditionally considered a marker of aging, mLOY has recently been linked to various pathologies including cancer, Alzheimer’s disease, and cardiovascular disease [109]. Recently, an mLOY polygenic risk score was associated with worse ISFO (3-month mRS) [137] (Fig. 5).

## Conclusions

Diverse biological processes are involved in poststroke recovery. Genetic alterations in pertinent systems can influence functional outcome as described by metrics such as mRS. Significant polymorphisms have been identified in genes related to the eicosanoid inflammatory pathway including those encoding cytochromes, cyclooxygenase, epoxide hydrolase, prostacyclin synthase, and thromboxane-A synthase. Altered proteins related to innate immunity including toll-like receptors, interleukins, C-reactive protein, and myeloperoxidase also affect IS outcome. Noncoding RNA variants regulating cytokine signaling are relevant as well. Proteins involved in primary hemostasis like GPIIIa and P2Y1 are vulnerable to genetic mutations affecting this phenotype, as are atherosclerosis-related proteins such as angiotensin converting enzyme. Significant variants have been found at the APOE, BDNF, and IGF1 loci, indicating that appropriate lipid metabolism and normal growth factor behavior are both crucial for poststroke recovery. Members of the HPT axis including dioxygenases and organic anion transporters have also been implicated. Several elements of the p53 pathway affect poststroke disability as well as various mitochondrial perturbations; markers of structural imbalance such as CNV and mLOY are among the most recently discovered effectors of stroke outcome.

**Fig. 6:**
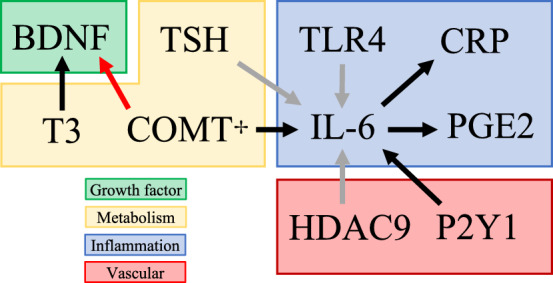
Demonstrates potential interconnections between some of the biological systems implicated in ISFO. Arrows from one component to another are color-coded: black represent induction, grey indicates NF-κB signaling, and red denotes negative regulation. ✢: decreased COMT, specifically, induces IL-6 release

Although these systems seem disconnected, links between different pathways may exist (Fig. 6). For example, IL-6 stimulates CRP secretion [121]. Thus, stroke recovery in patients with only the relevant IL-6 polymorphism (rs1800795) may have additional risk insofar as differential IL-6 levels could functionally mimic CRP rs1130864 which increases CRP levels in Han Chinese populations and leads to worse outcomes. In addition, there is evidence of IL-6-dependent secretion of prostaglandin E2 (PGE2)—a member of the cyclooxygenase pathway—in inflammatory states [28]. IL-6 also activates the hypothalamic–pituitary–adrenal axis which interacts with the HPT axis, so similar connections may exist between those systems as well [60, 211]. Further complexity can be postulated given that other inputs, such as P2Y1 receptor signaling [92] or decreased COMT activity [114], induce IL-6 release. As a result, changes in primary hemostasis or catecholamine metabolism may alter cytokine activity and thus function as a proxy for genetic variants in multiple systems [115]. TLR4 activates NF-κB signaling which stimulates the release of multiple inflammatory cytokines including IL-6 which may explains its large effect size on ISFO [177]; HDAC9 and TSH activate NF-κB signaling as well [10, 15]. BDNF also exemplifies the interconnectedness of these systems. Significant interactions between BDNF *val66met* and COMT *val158met* were reported in a study examining cortical plasticity [292]. Mechanistically, this can be explained by the fact that COMT *val158met* increases dopamine levels in the synaptic cleft [291] and that dopamine receptor activation increases BDNF expression [290]. There is also evidence that BDNF is induced by T3, suggesting a connection with the HPT axis [261].

Studies investigating the genetics of ISFO have mostly utilized a candidate gene approach for which preselected variants are examined for associations with various outcome metrics. The limitations of this methodology include selection bias, high false positive rate, and low genetic coverage [63, 80]. Considering most studies related to this topic have employed a candidate gene approach, reported findings should be interpreted with appropriate caution. In 2017, the Genetics of Ischaemic Stroke Functional Outcome (GISCOME) network was established to pool genotypic and phenotypic data from multiple institutions to foster large-scale, genome-wide association studies [187]. Söderholm et al. was the first GWAS examining functional outcome using the GISCOME dataset and found a single significant intronic variant (rs1842681) which is not obviously related to polymorphisms previously identified by candidate gene studies. Mola-Caminal et al. also found a novel locus, PATJ [197], and aside from PATJ and IGF1 both contributing to the PI3K-Akt signaling pathway, there is no clear connection to prior genetic variants of interest. As shown here, no loci identified through candidate gene studies have been recapitulated by the recent GWAS which may indicate marginal significance of canonical variants, highlight the heterogeneity of population-specific associations, or reflect the shortcomings of a candidate gene approach. Cross validation experiments as seen in Zhu et al. [332] where suggestive GWAS findings were confirmed using a candidate gene approach are necessary to reinforce suspected variants.

Overall, our search identified 74 genetic variants spanning 48 features are associated with ISFO. Most variants led to worse functional outcome, while nearly twenty variants appear to have protective effects (Supplemental Table 1, 2, 3, 4, 5, 6, 7). Within a single locus, some SNPs may worsen outcome, while others improve it. No pattern is observable, it simply depends on what mechanisms are altered. Among studies that used mRS as an outcome metric, SNPs of UCP (OR = 0.05), IL-6 (OR = 0.1), and TUG1 (OR = 0.499) were the most protective, while variants at the TLR4 (OR = 14.16), CRP (OR = 4.70), and TP53 (OR = 3.89) loci increase risk the most (Fig. 5).

While the literature regarding ISFO has significantly expanded in recent years, further investigation is required to verify the genetic variants discovered thus far and elucidate connections between different biological systems. For precision medicine to become practical in poststroke care, polymorphisms associated with functional outcome must be inventoried and described in a granular fashion. Aggregating and summarizing the variants of interests will allow investigators to take the next steps: comparative analysis to determine the weighted influence of each polymorphism, genotype-guided treatment trials, and machine learning for outcome prediction.

### Review criteria

A generalized search was conducted in PubMed with the following terms: (stroke OR ischemic stroke) AND (genotype OR genetic variant OR polymorphism) AND (functional outcome). Publications that examined associations between genetic polymorphisms and ischemic stroke functional outcome metrics (see below) were aggregated. Pediatric studies were excluded. Each gene of interest was further investigated in PubMed using the formula, ([gene of interest]) AND (ischemic stroke).

### Outcome metrics

Several metrics exist to quantify and evaluate neurological functioning poststroke. Neurologic deterioration (ND) describes clinical worsening based on changes in impairment scores denoted by the Glasgow Coma Scale (GCS) or the NIH Stroke Scale (NIHSS) [212, 243]. Recovery status is commonly examined using the modified Rankin Scale (mRS) and the Barthel Index (BI). mRS output is a discrete value ranging from 0 to 6, assessing a patient’s ability to perform basic tasks such as ambulation, with zero meaning no disability and six indicating death. The original Rankin Scale (RS) was only from 1 to 5. The asymptomatic and death categories were added later. [37] BI tests a patient’s ability to complete various functional tasks and is scored on a scale from 0 to 100; higher scores imply independence [221]. A modified Barthel Index (mBI) was developed to enhance specificity by increasing the number of scoring categories [239] Functional Independence Measure (FIM) is another method of examining functional status. It contains 5 cognitive and 13 motor sections. Each item scored is from 1–7 and low scores indicate increased dependence [173] The Rivermead Motor Assessment (RMA) specifically tests motor function [6] and the Fugl-Meyer Assessment is a quantitative index to assess motor function and some sensation qualities [91].

### Supplementary Information

Below is the link to the electronic supplementary material.

Supplementary file1 (DOCX 1102 KB)

Supplementary file2 (XLSX 11307 KB)
