# Supplementary material for: Genetics of ischemic stroke functional outcome
Source: J Neurol. 2024 Mar 19;271(5):2345–69. doi: 10.1007/s00415-024-12263-x (PMC11055934; doi:10.1007/s00415-024-12263-x)
Supplement: Supplementary file 1 — Supplementary file1 (DOCX 1102 KB) [file 415_2024_12263_MOESM1_ESM.docx]

**Supplemental Table 1.** A summary table of inflammation-related genetic variants associated with ischemic stroke functional outcome.

**Supplemental Table 2.** A summary table of vascular homeostasis-related genetic variants associated with ischemic stroke functional outcome.

**Supplemental Table 3.** A summary table of growth factor-related genetic variants associated with ischemic stroke functional outcome. ✢ indicates outcome score was calculated after specific rehabilitation efforts.

**Supplemental Table 4.** A summary table of metabolism-related genetic variants associated with ischemic stroke functional outcome. ✢ indicates outcome score was calculated after specific rehabilitation efforts.

**Supplemental Table 5.** A summary table of p53 regulatory pathway-related genetic variants associated with ischemic stroke functional outcome.

**Supplemental Table 6.** A summary table of mitochondria-related genetic variants associated with ischemic stroke functional outcome.

**Supplemental Table 7.** A summary table of GWAS-related genetic variants associated with ischemic stroke functional outcome.
